# Detecting and Quantifying Residual Intracardiac Shunts Using Oximetric Step-Up Methods: A Prospective Observational Study

**DOI:** 10.7759/cureus.33942

**Published:** 2023-01-18

**Authors:** Vishal V Bhende, Tanishq S Sharma, Ashwin S Sharma, Amit Kumar, Nirja P Patel, Hardil P Majmudar, Mamta R Patel, Kruti A Patel, Gurpreet Panesar, Kunal Soni, Kartik B Dhami, Sohilkhan R Pathan, Dushyant M Parmar, Paresh Nerurkar

**Affiliations:** 1 Pediatric Cardiac Surgery, Bhanubhai and Madhuben Patel Cardiac Centre, Shree Krishna Hospital, Bhaikaka University, Karamsad, IND; 2 Pediatric Cardiac Surgery, Gujarat Cancer Society Medical College, Hospital and Research Centre, Ahmedabad, IND; 3 Pediatric Cardiac Intensive Care, Bhanubhai and Madhuben Patel Cardiac Centre, Shree Krishna Hospital, Bhaikaka University, Karamsad, IND; 4 Cardiac Anaesthesiology, Bhanubhai and Madhuben Patel Cardiac Centre, Shree Krishna Hospital, Bhaikaka University, Karamsad, IND; 5 Biostatistics, Bhaikaka University, Karamsad, IND; 6 Perfusion Technology, Bhanubhai and Madhuben Patel Cardiac Centre, Shree Krishna Hospital, Bhaikaka University, Karamsad, IND; 7 Cardiac Anaesthesiology, Bhanubhai and Madhuben Patel Cardiac Centre, Shree Krishna Hospital, Bhaikaka University, Karmasad, IND; 8 Clinical Research Services, Bhanubhai and Madhuben Patel Cardiac Centre, Shree Krishna Hospital, Bhaikaka University, Karamsad, IND

**Keywords:** congenital heart diseases, pulse oximetry, hypoxemia, arterial blood gases, arterial blood oxygen saturation

## Abstract

Background & aims

Intracardiac shunts are abnormal channels of blood circulation within the heart that develop either as an additional blood flow pathway or as a replacement for the normal channels of blood circulation. They are the commonest types of congenital heart defects. Various methods are available in the present times to identify, localize or quantify left-to-right intracardiac shunts. Methods may vary in sensitivity, indicators, or types of equipment available. One such method used in almost all cardiac centers for a long time has been oximetry run to detect step-up differences in oxygen saturation values. In the oximetry run the main approach to detect and estimate the left-to-right (L-->R) shunts requires the oxygen concentration expressed as a proportion of saturation to be evaluated in blood samples which are obtained from the right atrium (RA) and pulmonary artery (PA), respectively. A left-to-right shunt can be considered if there is a significant increase (step-up) in blood saturation. A significant step-up is defined as a substantial rise in blood oxygen content or saturation that is higher than normal values.

Methods

Using a prospective observational design, this article investigates the application of the step-up method in detecting intracardiac shunts. The study was conducted between 2021 and 2022 on 35 pediatric cardiac patients (males/females, 24/11) diagnosed with post-tricuspid shunts. The pulmonary artery and right atrium were sampled before and after cardiopulmonary bypass surgery and analyzed using a blood gas test. As a result, nearly 91% of the patients had a saturation below 8%. However, the difference between PA oxygen saturation (SO_2)_ & RASO_2_ before and after surgery was significant. As a result, the difference in O_2_ saturation helped detect the residual ventricular septal defect (VSD) after the surgery.

Results

There were no deaths or complications in this study. There were no re-interventions for post-tricuspid shunt surgery, though one patient had a step-up of >15% and residual VSD status was moderate to large on two-dimensional (2D) echocardiography.

Conclusion

A combination of physical findings, chest radiography, electrocardiogram (ECG), and echocardiography is routinely done for all these patients undergoing pediatric cardiac surgery. Echocardiography can detect the occurrence of shunt but does not calculate the shunt ratio. Transesophageal or epicardial echocardiography is the standard of care but has its limitations like perception difference between the operating surgeon and the person performing echocardiography.

In this study, we have added an oximetry analysis of blood-gas samples before and after surgery and compared it to 2D echocardiography to test the validation of oximetry in isolation and comparison to 2D echocardiography.

## Introduction

A large number of people are affected by cardiovascular diseases including diseases of the heart and circulatory system; therefore, introducing effective diagnostic and monitoring methods is crucial for the well-being of these patients. In the normal body, blood flows in a unidirectional manner from the left to the right heart through the vascular bed of the circulation system. However, under pathological conditions, oxygen-rich blood takes a shorter path from the left to the right heart i.e., left-to-right shunts (L--->R shunt), without passing through the vascular bed. That could happen in many pathological cases such as atrial septal defects (ASD), ventricular septal defects (VSD), or patent ductus arteriosus (PDA). Congestive heart failure results from congenital causes as described above, or acquired causes such as myocardial infarction [1]. 

About 0.8% to 1.2% of births globally are associated with some type of birth defect. Shunts are divided into two types: cyanotic and non-cyanotic. The presence of a shunt can also affect left and/or right heart pressure [1]. The L--->R shunt occurs when the blood moves directly from the systemic circulation to the pulmonary circulation. This makes the pulmonary flow more important than systemic flow. Various methods are available at present to identify, localize, or quantify left-to-right intracardiac shunts. Methods may vary in sensitivity, indicators, or equipment available. Treatment of shunts depends on the type of condition and ranges from clinical observations like “watchful waiting,” medications, and lifestyle changes to surgery [2].

One such method that has long been used in almost all cardiac centers is the oximeter to detect differences in oxygen saturation. Oximetry has been used for more than five decades now to diagnose intracardiac and great-vessel shunts. In the oximetry run, the basic way to detect and estimate the L--->R shunt requires measuring the oxygen level or saturation of blood samples taken from the right atrium (RA) and pulmonary artery (PA), respectively. An L-->R shunt can occur following a substantial increase in oxygen content or if blood saturation is detected in any of the heart's chambers. It is often used to identify VSDs that persist after surgical repair in the operating room [3].

A significant step-up is defined as a rise in blood oxygen concentration or saturation beyond the normal values. If oxygen content in the PA is unexpectedly high i.e., a saturation >80%, then there is a likelihood of L--->R intracardiac shunt. This can help determine if the post-tricuspid shunt has not been treated properly and perhaps indicate a re-do surgery. If an increase in oxygen content in the blood sample from the distal side is detected at higher levels compared to the proximal chamber, then an L--->R shunt is assumed to have occurred. Due to differences in blood flow and rapidly changing physiological functions of blood and oxygen levels in the heart, increased oxygen levels may not reflect the true effect of residual VSD. In rare cases, L-->R and right-to-left (R-->L) shunt shunts could appear simultaneously in the same patients.

The effect of fluctuations in hemoglobin level can also be used to evaluate the step-up in saturation by comparing changes in the proportion of oxygen saturation and oxygen concentration. It was also observed that different values of percent saturations were known to help rather than changes in oxygen concentration for visualizing the cardiac shunt. For instance, VSD is diagnosed with a step-up of 5% between the right ventricle (RV) and PA.

Heparinized arterial blood gas (ABG) analysis is done by the addition of fluid. This statistically decreases the partial pressure of carbon dioxide (pCO2), partial pressure of oxygen (pO2), bicarbonate (HCO3), and base excess, while pH remains unchanged.

Echocardiography can also be used to detect the occurrence of a shunt but it cannot calculate the shunt ratio. This could be the critical factor that influences the treatment option. Contrast echocardiography studies have higher sensitivity and are very useful in diagnosing small to large L-->R shunts. Tomography or “thin section” imaging can be provided by two-dimensional (2D) echocardiography [4].

Transesophageal or epicardial echocardiography is preferred for care. However, it has its limitations. For quantitative and qualitative assessment of cardiac anatomy and features, transthoracic echocardiography (TTE) is an extensively available, reproducible, non-invasive imaging modality [4]. Transthoracic echocardiography is regularly used as a first-line cardiac imaging modality thanks to its extensive availability, non-invasiveness, and absence of radiation exposure.

The transesophageal echocardiogram (TEE) can be used to non-invasively compare stress gradients between heart chambers or between heart chambers and blood vessels. This allows the estimation of the intra-cavity pressure. It is expensive to purchase and additionally, there may be a perception difference between the operating surgeon and the person performing the echocardiography [5].

Rarely, deoxygenated blood can flow in the opposite direction of the L-->R shunt, taking the path from the right to the left heart. Eisenmenger's syndrome is one of these rare cases that can develop if mild or large VSD is not treated on time [6,7].

Detection of intracardiac shunt

The shunt can be diagnosed using invasive and noninvasive methods, including MRI in addition to Doppler echocardiography, and many others. Presently, many clinicians suspect the occurrence of shunts based on physical examination or electrocardiogram (ECG) along with the diagnosis on echocardiography followed by quantification via right-heart catheterization.

Shunts in the heart can be diagnosed using various methods, and subsequently localized and quantified through cardiac catheterization. Indicator dilution is a historical method limited to the research area and is not currently applicable in the practical field. This method involves the intravenous administration of agents like indocyanine blue, or its direct injection into the right heart. Later, the dye curve measurements generated from the continuous monitoring of the dye in the systemic circulation system provide a reasonable assessment of the shunt. However, the procedure is time-consuming and requires special instruments that are no longer available in catheterization laboratories. Another way to evaluate the shunt is contrast-enhanced angiography, which can be used to detect and diagnose VSD. In this method, the contrast agent is injected into the high-pressure chamber of the suspected shunt (such as the left ventricle)

Because of its simplicity, reproducibility, and availability in modern labs, measurement of oxygen saturation at various locations of oximetry or analysis of the venous system and right heart, is a frequently used invasive procedure.

## Materials and methods

Aims and objectives 

This study aimed to: 

· Detect and quantify residual intracardiac post-tricuspid shunt in pediatric patients undergoing cardiac surgery. Additionally, we aimed to compare the RA and PA saturation in pediatric patients who underwent cardiac surgery. This was done using samples of heparinized arterial blood gas pre and post-bypass (that were collected intraoperatively), and cardiopulmonary bypass (CPB) indices. 

· Analyze the usefulness of step-up measurement in detecting residual VSD post-operatively.

· Enhance literature on step-up analysis and its usefulness in present times.

**Figure 1:**
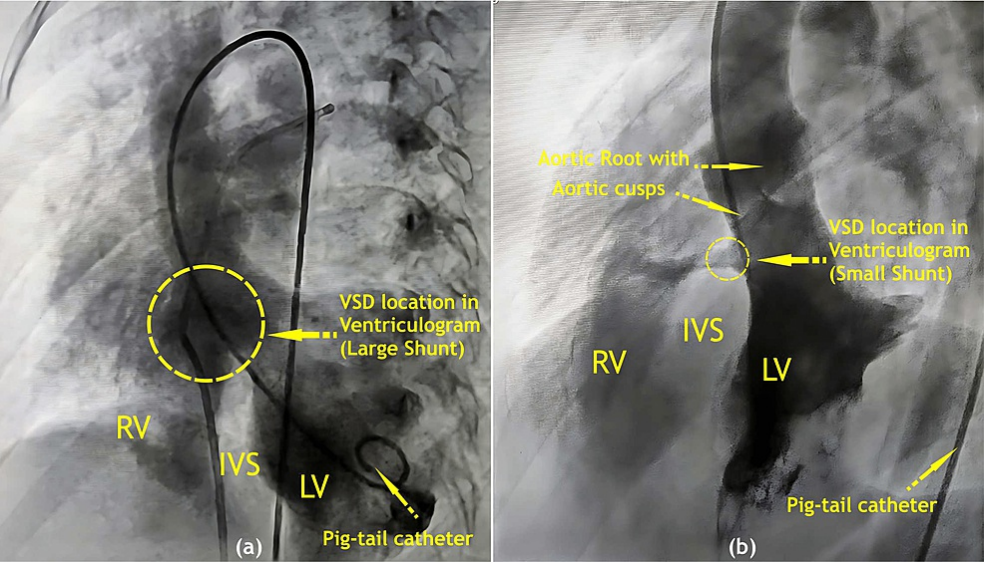
This cardiac ventriculography in LAO 60 degrees and cranial 20 degrees view, shows the dye flow from the left to the right ventricle through the VSD in (a) the large shunt, and (b) the small shunt. VSD: Ventricular septal defect, RV: Right ventricle, LV: Left ventricle, IVS: Inter-ventricular septum, LAO: Left anterior oblique

· Compare the usefulness of step-up over intraoperative echocardiography to detect significant residual VSD. Especially in pediatric heart centers that do not have full-time pediatric cardiologists to analyze 2D echocardiography, and who can instead, rely on this calorimetric testing for an assessment of residual VSD (Figure 1). 

**Figure 2:**
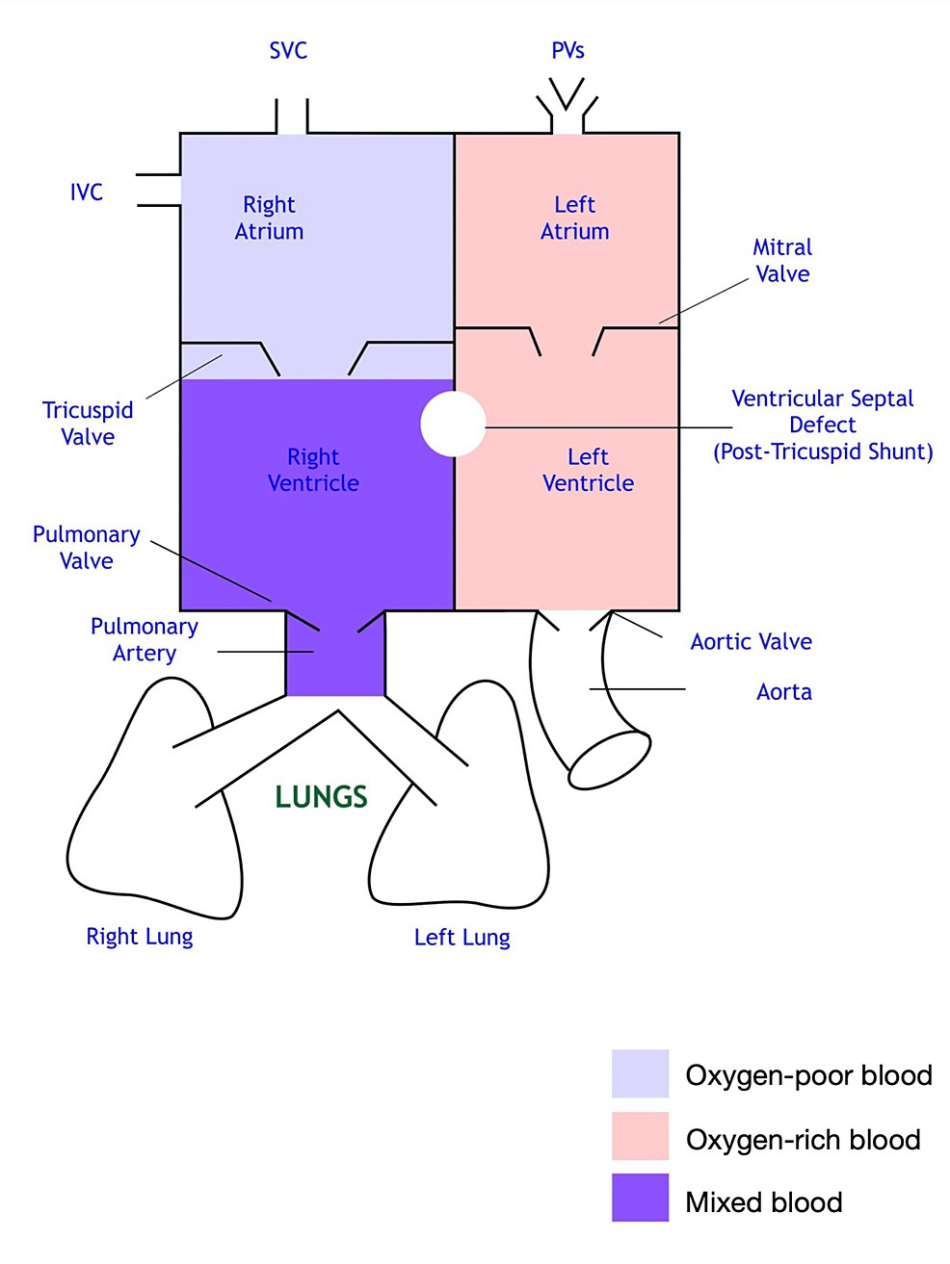
Mixed venous blood SVC: Superior vena cava, IVC: Inferior vena cava, PVs: Pulmonary veins

**Figure 3:**
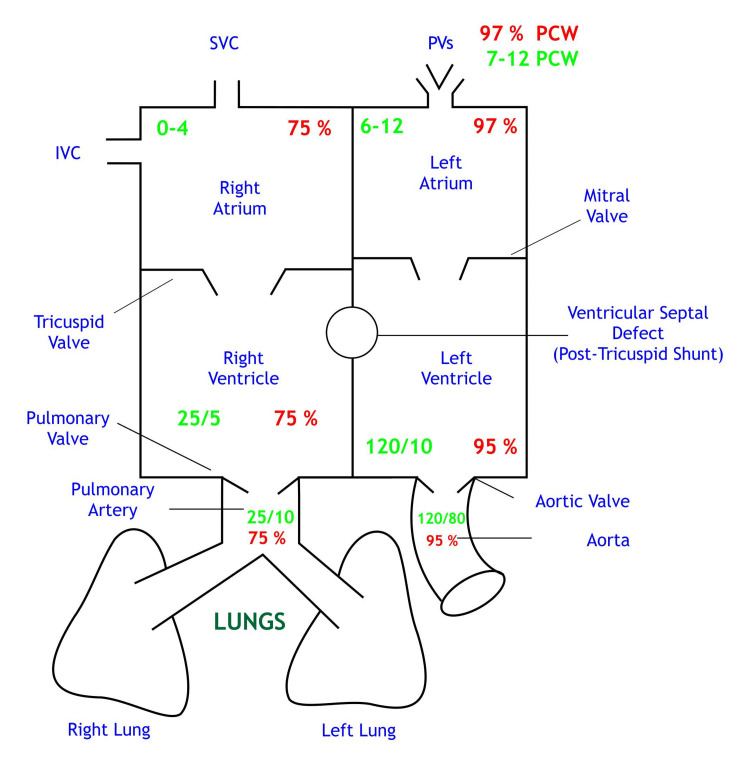
Normal pressures and O2 saturations Saturations are depicted in red; Chamber pressures are depicted in green SVC: Superior vena cava, IVC: Inferior vena cava, PVs: Pulmonary veins, PCW: Pulmonary capillary wedge

A basic mixed venous blood flowchart with normal pressures and O2 saturation physiology is shown in Figures 2, 3. 

Study population

This prospective observational investigation included pediatric patients (sample size n=35) who underwent CPB surgery at Bhanubhai and Madhuben Patel Cardiac Centre, Karamsad, Gujarat, India (Table 1).

**Table 1:** Master chart of patients enrolled in the study P: Patient,  CPB: Cardiopulmonary bypass, ACC: Aortic cross-clamp, Echo: Echocardiography, TOF: Tetralogy of Fallot, VSD: Ventricular septal defect, ASD: Atrial septal defect, RVOT: Right ventricular outflow tract, PAH: Pulmonary arterial hypertension, MS: Mitral stenosis, PS: Pulmonary stenosis, DORV: Double outlet right ventricle, PDA: Patent ductus arteriosus, CoA: Co-arctation of aorta, ICR: Intracardiac repair, AV: Atrio-ventricular, PFO: Patent foramen ovale, PM: Perimembranous, MR: Mitral regurgitation, MS: Mitral stenosis, BVF: Bulboventricular foramen, RPA: Right pulmonary artery, LV: Left ventricle, PR: Pulmonic regurgitation, RV: Right ventricle, RA: Right artery, PV: Pulmonary vein, PAPVC: Partial anomalous pulmonary venous connection

Sr. No.	Patient name	Age/ months	Sex	Weight (kgs)	Diagnosis	Surgical procedure	Preoperative echo	TEE/epicardial echo	Pre-bypass RASO_2_% PASO_2_%		Post-bypass RASO_2_% PASO_2_%		CPB time in minutes	ACC time in minutes	Re-intervention	Length of ICU stay (days)	Hospital stay (days)	Ventilator hours
1	P1	24	M	9.57	Restricted VSD	VSD closure	Restricted VSD (L->R shunt), LA & LV dilated, mild PAH	No residual VSD, good BVF	58	79	60	60	54	34	No	5	10	8
2	P2	6	F	4.1	DORV, VSD, MS, severe PAH	VSD closure + MV repair	Large VSD (L->R shunt), mild to moderate MS, LA & RV dilated, severe PAH	Small residual VSD (L->R shunt), moderate TR, mild MR, no MS, LA & LV dilated, good BVF	50	83	20	21	144	105	No	12	17	216
3	P3	3	M	5.99	ASD, VSD, PDA	VSD+PDA closure	Large VSD L->R shunt, small ASD, tiny PDA, severe PAH, good BVF	Dilated LV-LA, VSD patch seen in situ, no residual shunt seen, mild PAH	48	93	23	25	109	63	No	9	15	74
4	P4	96	M	17	Small to moderate perimembranous VSD, L->R shunt	VSD closure	LA & LV are dilated, no PAH	Mildly dilated LA/LV, VSD patch seen in situ, tiny residual VSD L->R shunt, no PAH, good BVF	70	85	69	70	58	41	No	2	7	10
5	P5	36	F	7.6	TOF	ICR	TOF with branch pulmonary arteries, malaligned sub-aortic VSD, severe PS, RA & RV dilated	Tiny VSD, no ASD, mild TR (gradient=40mm Hg), mild PR, good LV function	9	33	55	60	174	122	No	14	18	241
6	P6	144	M	26	VSD, RVOT obstruction	VSD closure + RVOT repair	Restricted perimembranous VSD L->R shunt, muscle bundle in RVOT, good BVF, no PAH	VSD patch seen in situ, good RV function, good LV function, no effusion	85	90	79	80	77	51	No	4	6	8
7	P7	7	M	3.7	Large PM VSD, OS ASD, small PDA, severe PAH	VSD closure with severe PAH	Large VSD, severe PAH, small PDA	Small VSD (gradient=39 mmHg), no ASD, moderate LV dysfunction, moderate PAH, no effusion	68	96	68	73	65	32	No	5	6	25
8	P8	7	F	5.2	ASD, VSD, moderate PAH	VSD closure	Small ASD (L->R shunt), VSD (L->R shunt), mild TR, good BVF	Dilated LV/LA, VSD patch seen in situ, no residual VSD seen, no PAH, mild TR	65	99	64	61	75	43	No	5	6	25
9	P9	7	M	3.2	VSD, MS, severe PAH	VSD closure + MV repair	Large VSD (L->R shunt), small ASD (L->R shunt), severe MS, severe PAH, LA/LV dilated	No residual VSD, no MS, muscle bundle in RV cavity, good RV & LV function	76	95	67	74	125	82	No	19	20	361
10	P10	4	M	4.22	Large VSD, PDA, severe PAH	VSD Closure	Large VSD (L->R shunt), small PDA (L->R shunt), mild MR, severe PAH	Dilated LA/LV, VSD patch seen in situ, small residual VSD (gradient =18 mmHg), mild MR, moderate TR, severe PAH	75	100	57	58	94	54	No	11	16	192
11	P11	12	M	6.8	Large ASD, large VSD, severe PAH	ASD + VSD closure	Large VSD (L->R shunt), mild MR, mild TR, severe PAH, good BVF	VSD patch seen in situ, 4.2 mm residual VSD (L->R shunt), VSD gradient=25 mmHg, no residual ASD, moderate TR	98	100	77	81	90	60	No	4	8	24
12	P12	12	M	12	ASD, VSD	VSD closure	Large VSD, mild PAH, mild AR, small ASD	VSD patch seen in situ, small residual VSD (L->R shunt), VSD gradient=44 mmHg, mild RVOT, good BVF	67	84	67	67	99	70	No	3	6	8
13	P13	9	M	7	Large VSD, ASD mild to moderate PAH	VSD+ASD closure	Large VSD, small ASD, LA/LV dilated, mild to moderate PAH	VSD patch seen in situ, no VSD, no ASD, good LV function	90	100	55	58	87	63	No	3	6	8
14	P14	12	F	4	Large VSD, severe MS, severe PAH	CoA repair + VSD closure, supramitral membrane resection	Large VSD (L->R shunt), no ASD, severe MS, mild to moderate coarctation of the aorta, severe PAH, good BVF	Mild MS, no MR, no shunt across the sub-aortic patch, muscular VSDs (L->R shunt), good LV & RV function, severe PAH	61	64	61	67	213	105	No	32	35	323
15	P15	24	M	9.7	VSD, ASD, mild AR, mild PAH	VSD + ASD closure	VSD, ASD mild AR, mild PAH	Dilated LA/LV, no residual VSD, no residual ASD, RVOT gradient=44 mmHg, mild TR, no effusion seen, good BVF	72	88	48	56	75	54	No	5	7	45
16	P16	12	M	7.3	Large ASD, VSD, severe PAH, Down's syndrome	VSD + ASD closure	Large subaortic VSD (L->R shunt), large OS ASD (L->R shunt) mild TR, severe PAH, good BVF	VSD & ASD patch seen in situ, no residual VSD seen, dilated RA & RV, moderate PAH, 2mm residual ASD, good BVF	96	98	50	46	95	64	No	5	10	24
17	P17	24	F	9.06	Mod VSD, mild to moderate PAH	VSD closure	Moderate PM VSD, mild to moderate PAH, good BVF	LVD: 3.9/3.3, EF: 30%, no residual VSD, no PAH, mild MR, mild TR	76	94	62	63	87	52	No	4	10	50
18	P18	60	M	15.2	VSD, mild AR, mild PAH	VSD closure	Restricted outlet VSD (L->R shunt), mild AR, mild PR, mild PAH, LA & LV dilated, good BVF	Trivial VSD, trivial AR, good LV function	84	87	76	76	90	64	No	3	7	7
19	P19	60	M	10.7	PM VSD, mild PAH	VSD closure	PM VSD (L->R shunt), aneurysm small IVS, LA /LV dilated, mild PAH	No residual VSD, no effusion, good BVF	80	95	71	67	65	40	No	3	8	7
20	P20	21	M	8.5	TOF with ASD	ICR	TOF with valvular & supra valvular PS with maligned double committed VSD, small OS ASD	No ASD, no VSD, no PS, free PR, severe TR, broad jet TR (gradient=30 mmHg), moderate RV dysfunction, good LV function	69	78	73	71	196	152	No	11	14	122
21	P21	36	F	7.5	Large VSD, severe PAH, nephrotic syndrome	VSD closure	large VSD (L->R shunt), mild TR, mild MR, mild to moderate PR, severe PAH, good BVF	Small residual VSD (gradient=38 mmHg), moderate to severe PAH, no TR, moderate MR, normal LV & RV function	50	90	63	79	81	47	No	6	9	25
22	P22	12	M	5.5	Large VSD, small ASD, severe PAH	VSD+ASD closure	VSD, ASD, severe PAH	Mod to large VSD(BD), aortic override, RV to AO patent, severe TR, LVOT: non-turbulent flow, moderate LV dysfunction, moderate PAH	71	86	14	42	136	82	No	10	12	145
23	P23	9	F	5	Truncus arteriosus of type 1	Truncus repair	Truncus arteriosus type 1, right aortic arch	LV hypertrophy, small residual VSD gradient=38 mmHg, mild AR, severe TR gradient=38 mmHg, mild LV systolic dysfunction	54	95	43	48	242	185	No	17	20	335
24	P24	12	F	6.5	TOF with PS	ICR	TOF with PS	VSD patch seen in situ, tiny VSD (R->L shunt), good LV function, TR not clearly assessed	14	22	51	53	174	131	No	10	13	143
25	P25	11	F	6.63	Perimembraneous VSD	VSD closure	Moderate PM VSD (L->R shunt), mild to moderate PAH, LA & LV dilated, good BVF	No VSD, sub-aortic spur, no AS, no AR, good BVF, no effusion	72	90	67	72	67	45	No	4	6	7
26	P26	12	M	7.5	TOF	ICR	TOF, malaligned sub-aortic VSD, severe sub- valvular	No VSD, no PS, mild TR, good RV & LV function	65	71	55	49	208	163	No	11	13	169
27	P27	120	M	21	TOF	ICR	TOF, small size branch PS, good BVF	Free PR, PS gradient=28 mmHg, no VSD, moderate TR, moderate RV dysfunction, normal LV function	24	86	85	86	192	134	No	8	10	49
28	P28	72	F	16.3	Moderate VSD	VSD closure	Moderate PM VSD, no PAH, no ASD, no PDA, good BVF	VSD patch seen in situ, good LV dysfunction, tiny residual VSD, moderate TR	72	89	90	75	72	41	No	3	6	7
29	P29	36	F	10.5	TOF, PAPVC	ICR, PAPVC re-routing	TOF, adequate size branch PAs, good BVF	Tiny VSD, mild PS gradient=22 mmHg, free PR, moderate TR gradient=40 mmHg, mild RV dysfunction	82	88	70	63	187	136	No	5	9	25
30	P30	24	M	9.86	Large VSD, severe PAH	VSD closure	large 11mm subpulmonic VSD, good BVF	No VSD, mild PR, no PS, mild PAH, TR gradient= 30 mmHg, good BVF	80	100	65	68	86	59	No	4	8	19
31	P31	10	M	6.4	VSD, severe PAH	VSD closure	Large VSD, moderate to severe PAH, good BVF	VSD patch seen in situ, tiny residual VSD, no AR, good BVF, mild TR gradient=18mmHg	82	99	54	49	81	51	No	5	9	11
32	P32	5	M	4.38	Down's syndrome, PDA, VSD	VSD closure + PDA ligation	Large VSD, PDA (L->R shunt), severe PAH	Tiny residual VSD (L-> R shunt), mild AR, mild MR, severe LV dysfunction	63	90	49	41	95	56	No	6	10	27
33	P33	24	M	10.5	Moderate VSD, moderate PAH	VSD closure	Moderate VSD, moderate PAH	VSD patch seen in situ, tiny residual VSD (L->R shunt), good BVF, mild TR (gradient=16 mmHg)	82	89	54	60	59	40	No	4	7	12
34	P34	6	M	3.8	Balanced complete AV canal defect, severe PAH	AV canal defect repair	Balanced AV canal defect, large ASD, inlet VSD, common AV valve, severe AV valve regurgitation, severe PAH	No VSD, PFO (BD), no MR, moderate to severe LV dysfunction	90	94	63	58	155	111	No	15	18	242
35	P35	156	M	20	TOF, absent PV, hemitruncus anomaly	ICR, hemitruncus repair	TOF, absent PV, severe PS, severe PR	Small residual VSD (gradient=32 mmHg), dilated RA & RV, small RPA origin, mild PR, no PS, fair LV systolic function	76	98	46	56	165	113	No	4	5	48

The research proposal was discussed and approval was obtained from the Institutional Ethics Committee of H.M. Patel Centre for Medical Care and Education, Bhaikaka University (approval no. IEC/BU/129/Faculty/1/206/2022). This study was conducted between June 2021 to June 2022.

Inclusion criteria

The study included patients with congenital heart disease and grown-up congenital heart disease as well as those with post-tricuspid shunts.

Exclusion criteria

Patients with pre-tricuspid shunts, single ventricle physiology, arch anomalies, and those that had a re-do surgery and require an emergency CPB, were not included in the study. 

Anesthesia protocol

All patients included in the study had a standardized anesthetic technique according to the protocol of our cardiac anesthetic department. Anesthesia was induced with fentanyl (1 to 2 µg/kg), ketamine (0.5 to 1 mg/kg), midazolam (0.02 to 0.03 mg/kg), vecuronium (0.08 to 0.1 mg/kg), and glycopyrrolate (2 to 4 µg/kg) and maintained with sevoflurane and top up doses of fentanyl, midazolam, and vecuronium. The standard monitoring included central venous pressure, invasive arterial pressure, and femoral arterial monitoring in addition to transoesophageal echocardiography.

Method

Cardiopulmonary bypass was initiated after heparinization to achieve a target-activated clotting time of greater than 480 seconds with intermittent intravenous heparin administration during the surgery. Non-pulsatile CPB was performed using a membrane oxygenator, namely the Terumo Capiox Baby FX 05 (Terumo Cardiovascular Systems Corp., Ann Arbor, MI, USA), Terumo Capiox Baby RX 05, and Dideco Lilliput D 902 (Dideco Lilliput Systems, Sorin, Italy), with an arterial filter (Baby Sherlock) and a roller pump. The oxygenator to be used is determined by the metabolic needs of the subjects which are usually related to age, body size, and body composition.

The blood samples to localize a step-up within the heart are received using an oximetry run. This study is technically performed on diagnostic oximetry runs, oxygen measurements, and blood flow calculations. The ABG test uses blood obtained from an artery where both the oxygen and carbon dioxide concentrations in addition to acid-base (pH) levels can be measured. This measures the pressure of oxygen dissolved in the blood and how well oxygen can move from the airspace of the lungs into the blood. This test requires a 2ml sample of blood from any of the following locations: the mid-portion, high point, or low or close to the tricuspid valve in the RQ; left and/or right PA; main PA.

Time of Sampling

**Figure 4:**
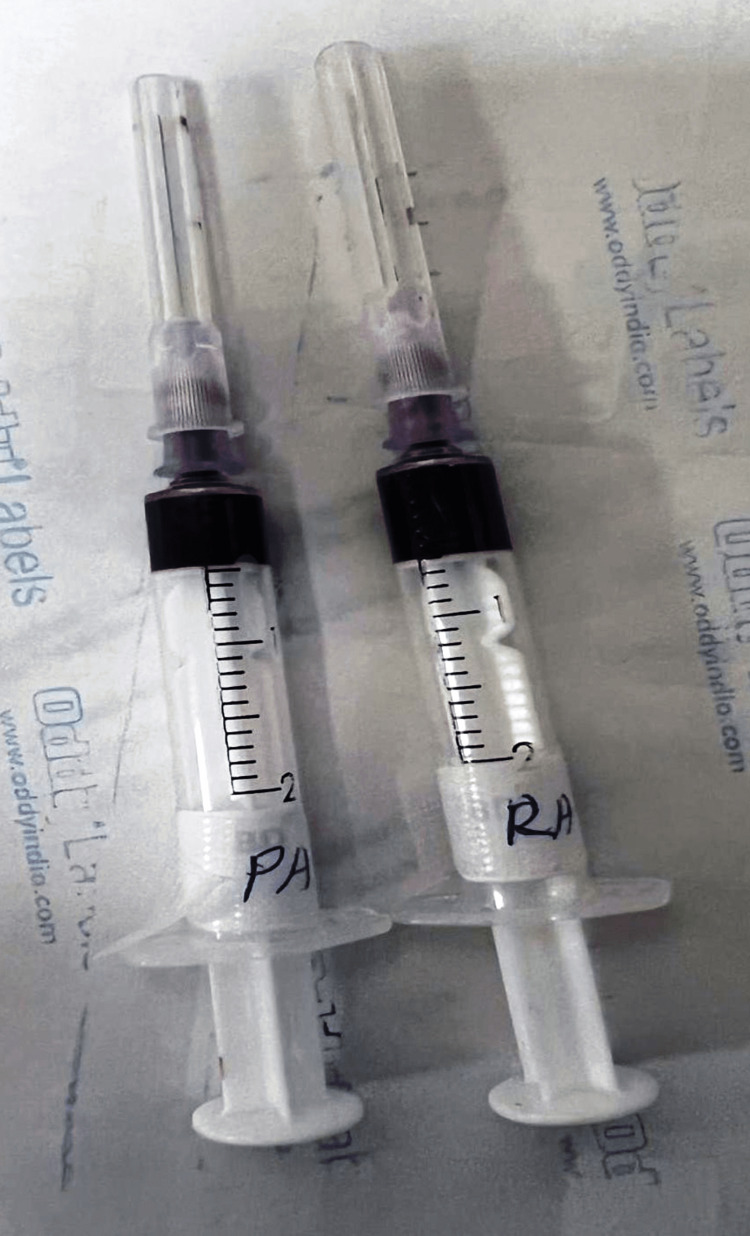
Pre-bypass right atrium (RA) and pulmonary artery (PA) samples

**Figure 5:**
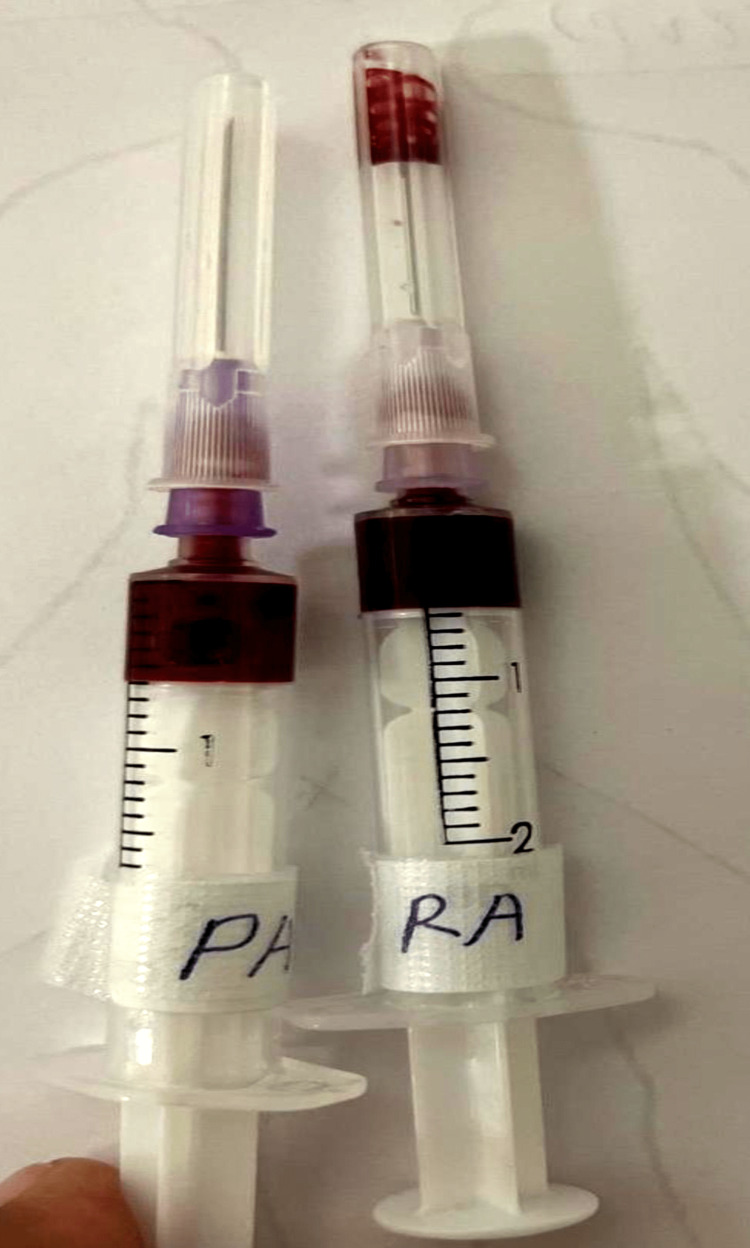
Post-bypass right atrium (RA) and pulmonary artery (PA) samples

The first sample of RA and PA was obtained just before the initiation of the bypass (Figure 4). The second sample of RA & PA was obtained after completing conventional ultrafiltration (CUF) and modified ultrafiltration (MUF), respectively (Figure 5). The second sample collection was executed after keeping a fraction of inspired oxygen (FiO2) of 50%=PaO2=0.5 x 500=250mmHg by adjustment of flows of O2 and air in the flow meter of the anesthesia machine.

Pre and post-bypass RA and PA samples were taken from the above-mentioned locations. The blood sample was then analyzed through a transportable device or at an on-site laboratory. The sample was analyzed within 10 minutes of the procedure to ensure an accurate test result by using a standard ABG analyzer (GEM Premier 3000 Blood Gas Analyzer, Wondfo Biotech Co., Ltd., Guangzhou, China) which is set to give a reading at a corrected temperature of 37oC. After the sampling, the RA saturation and PA saturation were noted down, and the pre and post differences were carried out. This test provided a precise measurement of oxygen in the body.

The findings from a blood gas test can help diagnose different diseases and also monitor response to treatment. The primary outcome variable of the present study was to find out the difference between the RA saturation and PA saturation pre and post-bypass in pediatric cardiac surgery done intraoperatively on CPB. The secondary outcome variable was the CPB time, aortic cross-clamp time (ACC), ventilator hours, and hospital stay. The O2 saturation step-up in this study is classified as follows: 8% to 10%=low, 11% to 15%=moderate, and 16% and above=high.

## Results

A total of 35 patients completed this study. Out of which, 24 were male and 11 were female (Table 2). The mean age was 32.24 months with a median (quartile 1 (Q1): nine months, Q3: 36 months) age of 12. The average weight of the patients was 9.09 kg and the median (Q1: 5.2 kg, Q3: 10.5 kg) weight was 7.5 kg. In the 35 patients with VSD, we describe three different percentage ranges under which there are <8, 9-10, and >15 O2 saturation, respectively. Thirty-two patients with a range of <8 O2 saturation between RA and PA had a valid 91.4% frequency. The O2 saturation range which lies between 9-10 had a frequency of 1 (2.9%). There were only two (5.7%) patients who had more than >15 difference in O2 saturation between RA and PA (Table 3).

**Table 2:** Demographic details of patients' sex

Sex	Frequency (%)
Male	24 (68.7%)
Female	11 (31.3%)
Total	35

**Table 3:** Oxygen saturation description O2: Oxygen

O_2_ saturation (%)	Frequency (%)
<8	32 (91.4%)
9-10	1 (2.9%)
11-15	0
>15	2 (5.7%)
Total	35

The preoperative minimum difference between RASO2 and PASO2 observed was 2 and the maximum was 62 while the post-bypass saturation minimum difference between RASO2 and PASO2 was -15% and the maximum was 28%. The preoperative mean (SD) difference between RASO2 and PASO2 was 18.65 (13.8) after surgery it was found to be 1.8 (7.3), which was statistically significant (p-value <0.001 using paired t-test). The median of the difference between RASO2 and PASO2 before surgery was 17 (the first quartile was seven, and the third quartile was 25) and after surgery, the median was 1 (the first quartile was a negative value of three, and the third quartile was five) with p-value <0.001 (using Wilcoxon signed-rank test), which was also statistically significant.

The number of VSD closures was 15. The mean (standard deviation (SD)) aortic cross-clamp (ACC) time was 47.5 (10.7) minutes, and the CPB time was 75.5 (14) minutes. These patients had a mean (SD) ventilator hours of 27.5 (46.9), an ICU stay of 4.46 (2.1) days, and a hospital stay of 8 (2) days. Patients with ICR were 7 with a mean (SD) CPB time of 185.1 (14) minutes, ACC time of 135.8 (17) minutes, ventilator hours of 113.8 (78), and a hospital stay of 11.7 (4.2) days (Table 4).

**Table 4:** Statistical data of operative characteristics SD: Standard deviation, VSD: Ventricular septal defect, ICR: Intracardiac repair, ASD: Atrial septal defect, PDA: Patent ductus arteriosus, CPB: Cardiopulmonary bypass, ACC: Aortic cross-clamp

Type of surgery	No.of patients (n)	CPB Time (minutes) Mean (SD)	ACC Time (minutes) Mean (SD)	Length of ICU stay (days) Mean (SD)	Ventilator (hours) Mean (SD)	Hospital stay (days) Mean (SD)
VSD closure	15	75.53 (14.02)	47.53 (10.7)	4.46 (2.09)	27.53 (46.9)	8.06 (2.63)
ICR	7	185.14 (14.97)	135.85 (17.02)	9 (3.55)	113.85 (78.06)	11.71 (4.15)
ASD + VSD closure	5	96.6 (23.22)	64.6 (10.47)	5.4 (2.70)	49.2 (55.14)	8.6 (2.40)
VSD + PDA closure	2	102 (9.89)	59.5 (4.94)	7.5 (2.12)	50.5 (33.23)	12.5 (3.53)
Others	6	159.33 (59.87)	106.5 (44.42)	16.5 (9.22)	247.5 (130.09)	19.33 (9.28)

**Figure 6:**
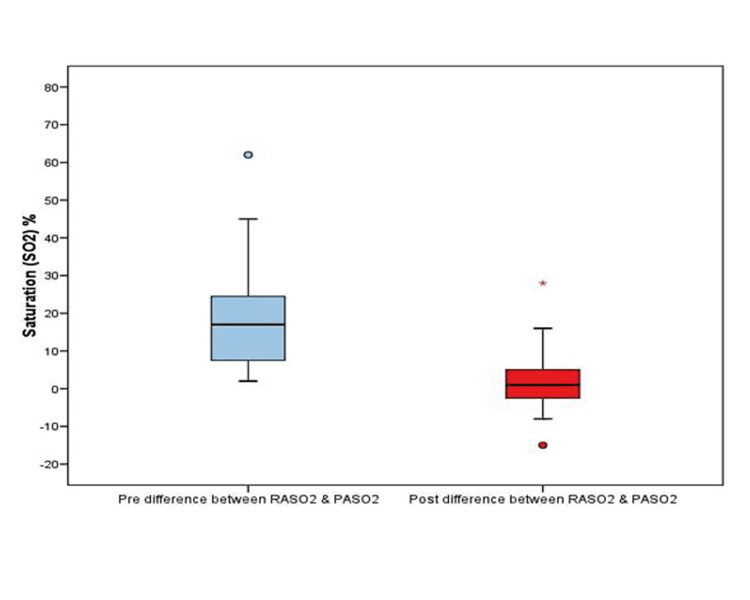
Shows the value of pre and post-bypass saturation differences between RASO2 and PASO2 RASO2: Right atrium oxygen saturation, PASO2: Pulmonary artery oxygen saturation

The distribution of the oxygen saturation difference between RA and PA is shown in Figure 6. On average, the PA saturation pre-bypass was higher than RA saturation.

**Figure 7:**
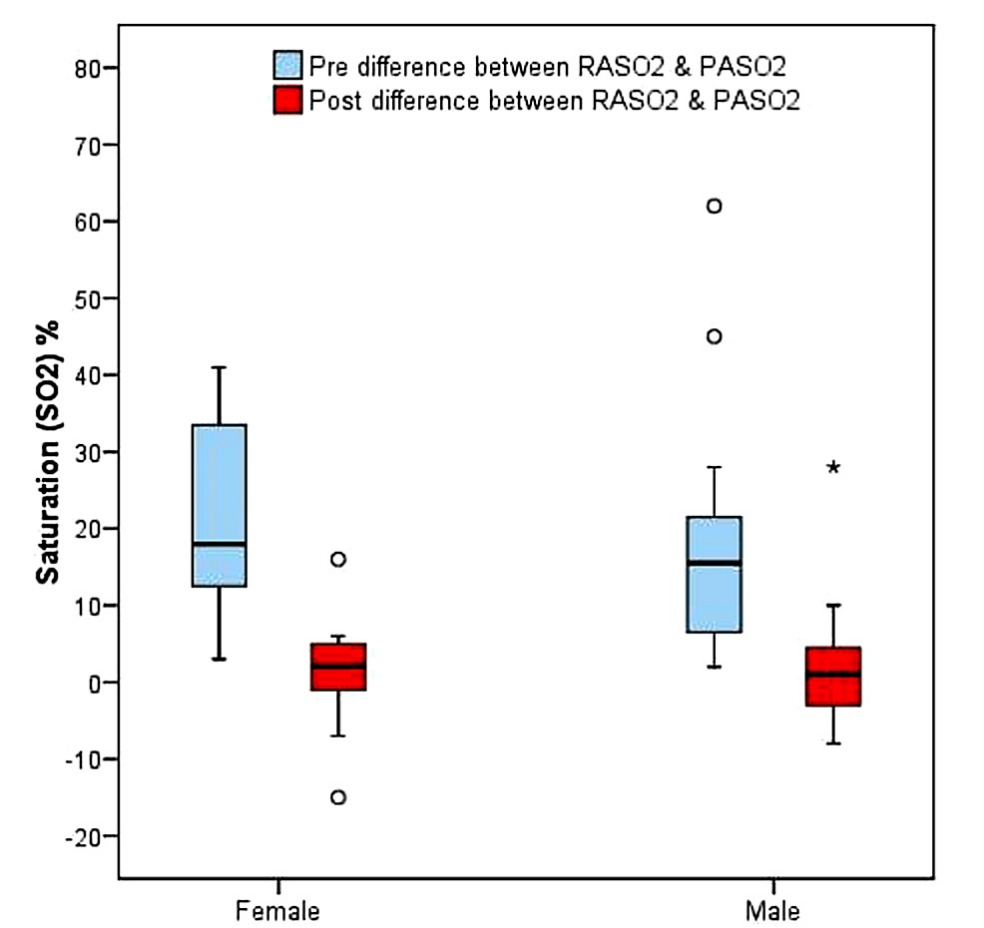
The value of pre and post-bypass saturation differences between RASO2 and PASO2 in females and males RASO2: Right atrium oxygen saturation, PASO2: Pulmonary artery oxygen saturation

The below box plot (Figure 7) shows the saturation comparison between females and males pre and postoperative. The median (Q1, Q3) pre-saturation difference between RASO2 and PASO2 in females was 18 (Q1=8, Q3=34) and in males, the median was 15 (Q1=8, Q3=20). The median post-saturation differences between RASO2 and PASO2 in females was 2 (Q1=-4, Q3=5) and in males, the median was 1 (Q1=0.6, Q3=3.3). 

**Figure 8:**
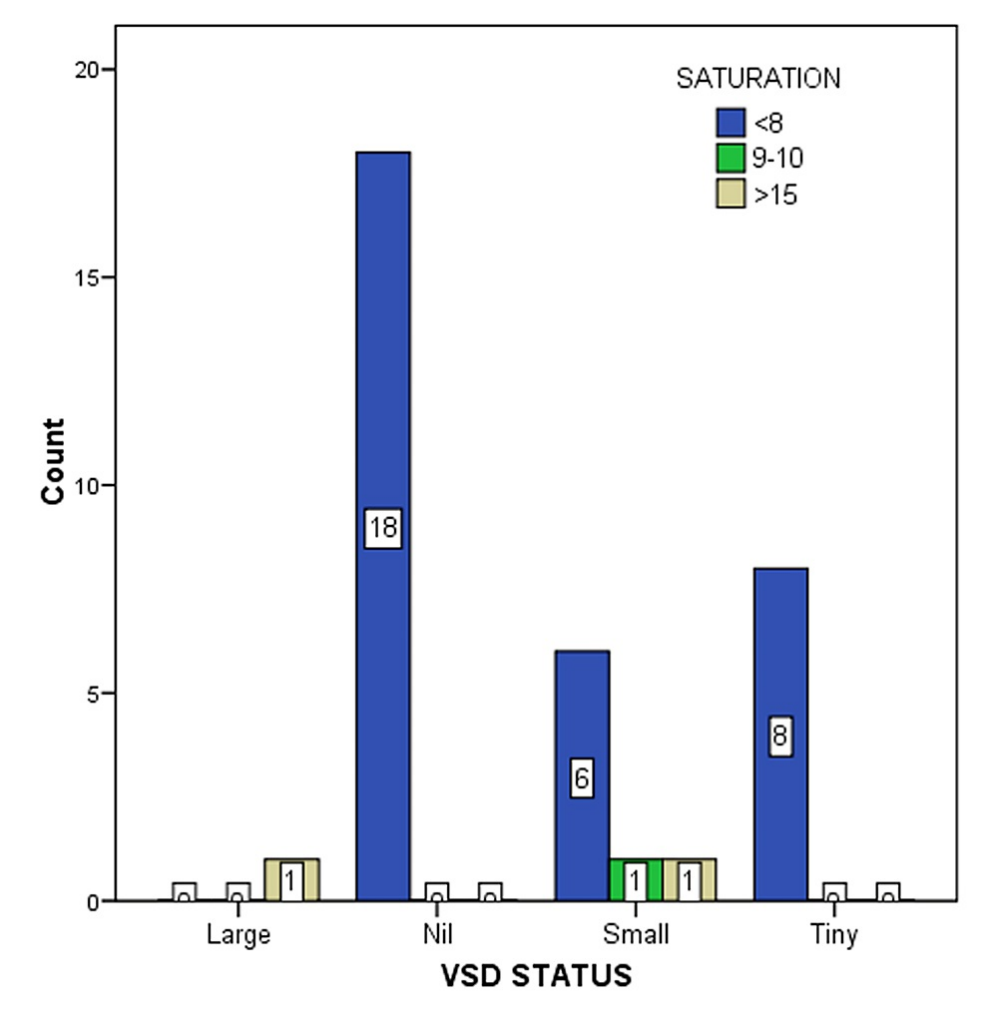
Correlation between residual VSD status on oximetry and 2D echocardiography VSD: Ventricular septal defect

When we calculate the possibility of successfully detecting a shunt, we utilize data based on echocardiographic findings only even though diagnosing the shunt can be guided by data from several other parameters like oximetry, heart sounds, hemodynamic recordings, angiocardiography, etc. (Figure 8), (Table 5).

**Table 5:** VSD status and saturation cross-tabulation count VSD: Ventricular septal defect

		Saturation			Total
		<8	9-10	>15	
VSD_status	Large	0	0	1	1
	Nil	18	0	0	18
	Small	6	1	1	8
	Tiny	8	0	0	8
Total		32	1	2	35

One patient had a large residual VSD. As expected, the saturation step-up was more than 15%. Another patient (12.5%) had a small residual VSD but had a significant step-up. It appears significant but as the total number is small (eight patients), a large study will be better to look for statistical significance. On the other hand, no significant step-up almost ruled out large residual VSD.

Statistical analysis 

All patient data were entered into Microsoft Excel. Data analysis was carried out using Stata version 14.2 (StataCorp, College Station, TX, USA). Continuous and categorical variables were reported using mean (SD), median (Q1, Q3 ), and frequency (%). To compare the results, we used the Student's t-test (continuous variable) and Wilcoxon Mann-Whitney test (categorical variable). A p-value of <0.05 was considered the cut-off value for the statistically significant test.

## Discussion

The ideal management for patients with VSDs would be a single-stage repair with complete VSD closure and avoiding complications such as heart block, arrhythmias, and ventricular dysfunction. The efficacy of the surgery is dependent on early operative risk, septal function after surgery, and the rhythm status. However, the morbidity and mortality related to VSD treatment remain higher than in other types of cardiac surgery. There were no early or late mortality and morbidity in the patients. Of the 35 patients with an intracardiac L-->R shunting, the PA saturation was higher than RA preoperatively. The saturation difference between RA and PA postoperatively suggests the mixing of blood from LV to RV shows that still a small to large VSD is present.

In our study, the RA blood samples were drawn from the mid-lateral portion of the RA, which is crucial because its saturation is minimally affected by inferior vena cava or coronary sinus blood. On the other hand, blood samples from PA were drawn from the pulmonary trunk. Also, in our study, one patient with a large VSD had a step-up of >15%, and another patient with a small residual VSD had a significant step-up of >15%. In the remaining 32 patients, no significant step-up was observed. As this study did compare data of oximetry with 2D echocardiography, the errors in missing out on correctable residual lesions diminished vis-a-vis performing the oximetry as a standalone study. In the first two patients who had residual VSD and step-up of >15%, no surgical re-intervention was done.

Left-to-right shunt leads to a rise in the oxygen level in the RV. However, even in normal and healthy people, it was quickly recognized that there was a discrepancy in the measured oxygen levels in the right heart. This variation was thought to occur from incomplete mixing of venous flow, assessment errors, and changes in the physiological status of the patients [1]. Several classic studies have been carried out to clarify the nature and size of this variability of oxygen levels in healthy individuals and define the criteria for the diagnosis of intra-cardiac shunts. The majority of the studies were carried out according to the Van Slyke method of directly assessing the oxygen content, however, the majority of catheterization laboratories at present utilize reflectance oximetry in estimating oxygen saturation. Therefore, new criteria for the diagnosis of shunt based on oxygen saturation levels are needed. Our study describes such criteria for pediatric subjects [2].

The RA receives input from three sources: the superior vena cava, the inferior vena cava, and the coronary sinus. However, the variation in the oxygen saturation is resulted from incomplete venous mixing, making RA a poor choice to truly estimate oxygen saturation. Therefore, the dependence on the results of only one or two samples to estimate oxygen increase is highly susceptible to error [8,9]. On the other hand, the variability in PA measurement is always attributed to inherited measurement errors. On the other hand, there is a normal increase in oxygen tension that may occur between successive right heart chambers even in the absence of an L-->R shunt. The abnormally elevated values in the RA sample may result from the intrinsic variation within the chamber (for example, one cause may be obtaining a sample from a venous stream having a greater level of oxygenated blood). There are two approaches to estimating these variations in the right chambers. The first is based on comparing the highest saturation values in the chambers, while the second compares the mean oxygen saturation values. [10] The first method leads to a smaller difference between values than the second. However, we have used the second approach in our study.

Two-dimensional echocardiography allows visualization of intra-cardiac communication with L-->R shunts. It provides excellent visualization of the inter-ventricular septum as well as color flow mapping which allows the detection of large L-->R shunts in VSD patients. This approach is highly sensitive in locating VSDs; either congenital or acquired [11,12]. However, 2D echocardiography has limitations in correctly quantifying the range of shunts from left to right but can give estimates of their size. For instance, a large VSD with an L--->R shunt may lead to right ventricular enlargement with diastolic septal flattening. This pattern is easily detectable by 2D echocardiography [13,14]. Large L-->R shunt VSDs usually cause an overload of right ventricular pressure, resulting in hypertrophy and flattening of the systolic and diastolic septum [14].

In Doppler color flow imaging, the size of the flow is relative to the shunt size. Trans-thoracic or TEE can detect small colorful jets generated by L-->R shunt in VSD cases, even if the oximetry results are negative [15]. Echocardiography is technically inadequate in some subjects, particularly in individuals having obesity, chronic lung disease, and chest wall deformity, so detecting, locating, and/or quantifying intra-cardiac shunts from left to right may be difficult or impossible [16]. In our observational study, we employed the double-check method of oximetry with postoperative 2D echocardiography in all patients (as seen above in Table 1). There was no re-intervention after correcting the VSD.

It is necessary to highlight the limitations of the model in order to build a valid mathematical model for the system. First, the model excluded any physiological fluctuations in oxygen saturation due to the incomplete mixing of venous blood in the vena cava and RV, particularly in the RA. To isolate the oximeter error in consideration in the detection of the shunt, we excluded the cause of this well-known venous saturation variation, which has been widely studied by others. Second, when we calculated the possibility of successfully detecting a shunt, the diagnosis was made completely without oximetry, but we believe that saturation levels are the only information available [15]. Alternatively, other data like heart sounds, hemodynamic recordings, echocardiography, and angiography can support and confirm oximetry diagnosis. Third, the relative uncertainty regarding measurement error and its effects on the shunt's oxygen measurement diagnosis depends on the situation. For instance, oximeter errors could be a major challenge if they are not appropriately utilized in a subject breathing pure oxygen because the instrument does not assess dissolved oxygen [16].

Similarly, the crucial role of oximeter error is related to the stability of systemic circulation. Both the shunt according to Fick cardiac output measurements and oximeter diagnostics are known to be steady-state methods. Therefore, the manual recommends taking a blood sample as soon as possible for “running the oximeter” [17,18]. However, if systemic circulation fluctuates during the time the blood sample is taken, the venous saturation level can change significantly as the tissue regulates oxygen extraction to maintain oxygen supply. Fluctuations due to this flow of venous saturation during sampling time can artificially increase saturation without the occurrence of a shunt, or offset or exaggerate the rise caused by a true shunt. Therefore, under these undesired situations, the role of the oximeter error in diagnostic uncertainty can be insignificant compared to other errors arising from transient blood flow.

Therefore, oximeter errors play an important role in shunt diagnosis when routine precautions are considered towards lowering changes in systemic perfusion. In conclusion, this study has shown the use of oximetry run to detect the residual shunt is a useful and safer technique and can help in detecting the size of the shunt and oxygen saturation in RA and PA (Table 6).

**Table 6:** Oximetry studies undertaken by different researchers

Sr. No.	Authors	Year of publication	Patients (n)	Conclusion/results
01	Dexter et al. [19,20]	1947	44	Pioneered research into techniques for the practice of oxygen densitometers, explained normal variability, and provided a measure of a significant increase in oxygen solely for the measurement of blood oxygen content.
02	Antman et al. [21]	August 1980	23 patients without left to right shunt vs. 42 patients with a left to right shunt	Claimed that an average difference of at least 7% oxygen saturation change in location was required for prognostication of an atrial shunt and 5% oxygen saturation for a ventricular or great arterial shunt.
03	Pirwitz et al. [22]	1997	102 adults	Decided a satisfactory formulation for estimating the combined venous oxygen saturation degree with a mathematical mixture of SVC and IVC saturation ranges. No pediatric population data
04	Freed et al. [4]	1979	1121 cardiac catheterizations in children	The involved pediatric population reported that a greater step-up occurs in less than 1% of cases, making the presence of an intra- cardiac shunt highly likely.
05	Barratt-Boyes (&) Wood [23]	1957	n=26 healthy, ambulatory subjects	Proposed several blood samples be taken from each right-sided chamber and that the oxygen content data of each chamber be averaged before applying Dexter's criteria to determine whether there is an “increase” or not.
06	Rudolph (&) Cayler [24]	1958	n=1250 patients, 244 patients under three years of age	Found a pair of samples with a 10% increase in saturation from SVC to RA, a 7% increase in saturation from RA to RV, and a 5% increase in saturation from RV to PA. Using Rudolf and Kayler's criteria results in a 0.4% false positive rate for PA-SVC, 0.3% for PA-RA, and 1.3% for PA-RV. It should be noted that the observed step-ups below the diagnostic cut-off do not indicate the absence of an intra-cardiac shunt. Even a significant shunt cannot produce higher elevations that can be found without a shunt.
07	Van Slyke (&) Neil [25]	1924	---	Showed a 9% step up from SVC to PA, a 6% step up from RA to PA, and a 6% step up from RV to PA for all catheterizations without shunt from left to right. It suggests that it occurs in less than 1%. Most of the early publications dealt with the oxygen content measured by the method of Van Slyke and Neil (1924).
08	Nadas et al.	1977	n=1000 children	Published a joint study of congenital heart disease and analyzed data to determine the normal range of shunt-free right heart saturation differences, and to define false positive rates for various oxygen metrics for left-to-right intra-cardiac shunts
09	Cournand et al. [26]	1945	n=22	Atrial and ventricular samples were rapidly and sequentially taken in 22 ''normal'' patients

Limitations

This study is limited due to the nature of the sampling methods, either whilst procuring from the non-designated locations in the cardiac chambers and/or not adjusting ventilator settings to achieve a FiO2 of 50% at the time of second sampling post-surgery. 

## Conclusions

Step-ups in oxygen content may be preferable to increases in saturation, since an increase in content by a given amount is an unambiguous measure of a given size of the shunt, while increases in saturation differ not only due to the occurrence of the shunt but also with the concentration of total hemoglobin. Until extra records are to be had concerning the correctness of the gadgets that determine each oxygen level material and saturation, the diagnosis of the presence of shunts needs to be based on the premise of step-ups in saturation as opposed to oxygen content material. Taking the average values of two or more blood samples obtained from each site and utilizing an oximeter having an error of 1% substantially increases the possibility of arriving at an accurate diagnosis. With the currently available devices, oximetry could possibly detect saturation step-ups as little as 3.6% (17% shunt) and it is safe to use and cheaper. Oxygen saturation data compared to oxygen content data is preferred to detect the occurrence of a shunt. Oximetry testing is handy in the setting of a primary VSD closure wherein significant step-ups post VSD closure point to an additional shunt not manifested in the preoperative echocardiography, provided the primary repair of VSD is satisfactory. This is the unlimiting factor of this test.

This study gains significance as facilities for echocardiography in the postoperative period is not routinely available at centers conducting pediatric heart surgeries due to varied reasons. In our institution, we had a double-check method of echocardiography as well as oximetry.
